# Impact of mechanical unloading on the irisin precursor, FNDC5, in skeletal muscle of sheep as a large animal experimental model

**DOI:** 10.1093/jbmrpl/ziag057

**Published:** 2026-03-31

**Authors:** Clelia Suriano, Onofrio Daniele Terrevoli, Francesco Staffieri, Luca Lacitignola, Antonio Crovace, Luisa Pellicani, Michela Taurino, Silvia Colucci, Maria Grano, Alberto Maria Crovace, Graziana Colaianni

**Affiliations:** Department of Translational Biomedicine and Neuroscience, University of Bari, 70124 Bari, Italy; Department of Precision and Regenerative Medicine and Ionian Area, University of Bari, 70124 Bari, Italy; Department of Precision and Regenerative Medicine and Ionian Area, University of Bari, 70124 Bari, Italy; Department of Precision and Regenerative Medicine and Ionian Area, University of Bari, 70124 Bari, Italy; Department of Precision and Regenerative Medicine and Ionian Area, University of Bari, 70124 Bari, Italy; Department of Translational Biomedicine and Neuroscience, University of Bari, 70124 Bari, Italy; Department of Translational Biomedicine and Neuroscience, University of Bari, 70124 Bari, Italy; Department of Translational Biomedicine and Neuroscience, University of Bari, 70124 Bari, Italy; Department of Precision and Regenerative Medicine and Ionian Area, University of Bari, 70124 Bari, Italy; Department of Veterinary Medicine, University of Sassari, 07100 Sassari, Italy; Department of Precision and Regenerative Medicine and Ionian Area, University of Bari, 70124 Bari, Italy

**Keywords:** irisin, FNDC5, muscle atrophy, translational research, large animal model

## Abstract

Scientific evidence has demonstrated the involvement of irisin, a molecule produced by muscle contraction, in preventing the onset of musculoskeletal decline. We previously demonstrated that the expression of irisin and its muscle-derived precursor FNDC5 is affected by unloading in skeletal muscle of hindlimb-unloaded (HU) mice. Studies in the HU mouse model showed that treatment with irisin prevents muscle wasting and atrophy. In parallel, human studies documented a strong association between irisin/FNDC5 and muscle function. Therefore, the scientific community is committed to determining whether irisin may represent a promising clinical strategy for preventing and treating disorders caused by muscle inactivity, such as those affecting bedridden or elderly patients. In translational research from mice to humans, there are very few in vivo studies conducted on large animals. The sheep model is an excellent model for studying the musculoskeletal system due to its anatomical characteristics, which share similarities with humans, allowing for the reproduction of mechanical load distribution on the limbs. To provide translational knowledge on the progression of muscle atrophy, we evaluated the impact of unloading on the expression of FNDC5 and functional proteins of skeletal muscle in sheep. Our findings revealed a reduction of FNDC5 in the quadriceps muscle after 2 wk of immobilization. The FNDC5 decrease is accompanied by a reduction of myosin isoforms *MyHC1* and *MyHC2x*, and an increase in muscle fibrosis. Importantly, FNDC5 expression positively correlated with expression of myosins, implying that the irisin/FNDC5 system is a driving force for muscle function in this large animal model.

## Graphical Abstract

**Graphical Abstract:**
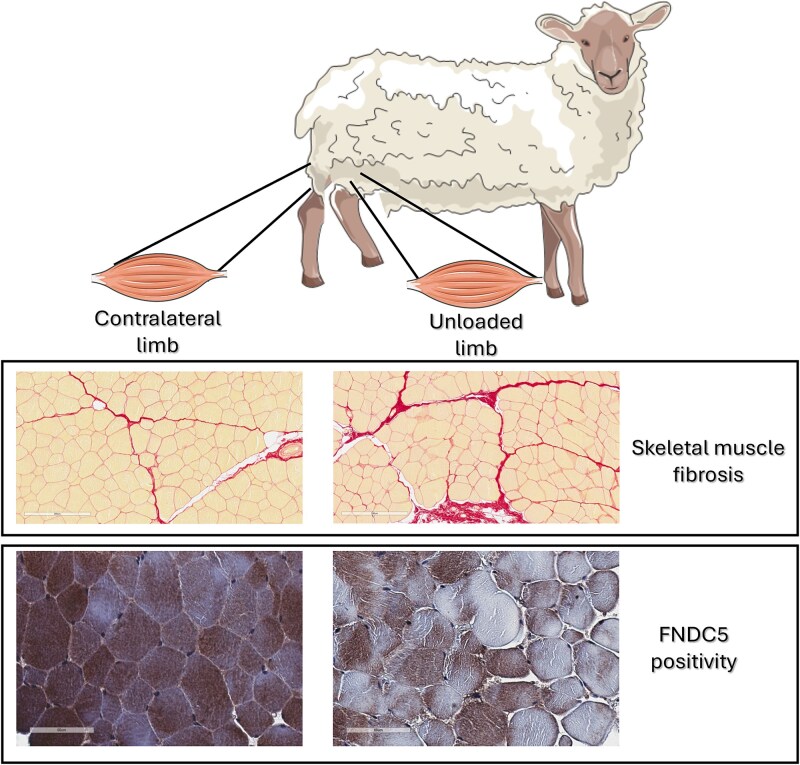

## Introduction

Skeletal muscle is a highly dynamic tissue that responds to various stimuli, especially changes in mechanical load. Consequently, the disuse of skeletal muscle directly affects the phenotype of muscle fibers, influencing morphological characteristics and protein expression.^1^ One of the muscle proteins that is a key load-sensitive molecule is the precursor of the myokine irisin, the transmembrane protein fibronectin type III domain-containing protein 5 (FNDC5).^2^ Irisin is produced by skeletal muscle during contraction, as a secreted portion of FNDC5.^3^

As evidence of the impact of disuse on circulating serum irisin and its muscle-derived precursor FNDC5, we previously studied the time-dependent effects of unloading conditions on muscle in unloaded hindlimb (HU) mice, demonstrating that FNDC5/irisin expression was strongly downregulated by unloading conditions, in parallel with decreased expression of the myosin isoform Type 2X-myosin heavy chain (*MyHC2x*).^1^ Consistently, studies in mouse models have shown that treatment with recombinant irisin prevents the onset of muscle atrophy caused by disuse or denervation.^4^^,^^5^

In humans, we and others have documented a strong association between irisin/FNDC5 and skeletal muscle functions.^6^^,^^7^ In a population of patients affected by Charcot-Marie-Tooth disease, a hereditary polyneuropathy characterized by progressive muscle degeneration, we observed that irisin serum levels positively correlated with muscle strength.^7^ Moreover, in a human model of simulated microgravity (Bed Rest), we found that individuals with high circulating irisin levels were protected from disuse-induced muscle atrophy compared to those with lower irisin levels.^8^ Finally, in a population of patients affected by myopathies, associated with muscle atrophy and necrosis of myofibers, the skeletal muscle expression of FNDC5 was significantly reduced compared to health controls, suggesting the involvement of the irisin/FNDC5 system in the disease pathogenesis.^9^

Considering the numerous preclinical validations in mouse models about the efficacy of irisin treatment in preventing musculoskeletal system damage caused by disuse, and in view of the results obtained from observational studies in humans, the development of an irisin-based pharmaceutical formulation could be an attractive future perspective. This type of drug therapy could be used in all patients in whom sedentariness, immobilization, or metabolic and neurodegenerative diseases result in severe decline of the musculoskeletal system.

In the transition from mice to human, there are very few in vivo studies conducted on large animals, such as the sheep model.^10^ This approach would provide key translational knowledge about disease progression and possible therapeutic mechanisms, which are critical for the development of new drug therapies in humans. Specifically, the sheep model is an excellent large animal model for studying the musculoskeletal system because of the anatomical and physiological features it shares with humans. Moreover, the sheep model mimics the distribution of mechanical loads on the limbs during loading activities, making it a highly comparable model to humans.^10^

In the present study, we evaluated over the period of 1-2 wk following immobilization the impact of the absence of mechanical loading on the expression of FNDC5 and functional proteins in quadriceps muscle of a sheep model. The results of this study showed that mechanical unloading caused a reduction of FNDC5 in sheep quadriceps, which was already evident after 1 wk, but became significant after 2 wk. The decrease in FNDC5 was accompanied by a significant reduction in the expression of myosin isoforms *MyHC1* and *MyHC2x* and an increase in muscle fibrosis. Notably, a positive correlation between FNDC5 and myosins was also observed, suggesting that the irisin/FNDC5 system is a driving force for muscle function also in this large animal model.

## Materials and methods

### Ethics statement and animal care

The present study was performed after approval by the Italian Ministry of Health (n° 771/2023-PR) (Smart Biomimetic Devices for Tendon Tissue Engineering) and in strict accordance with the recommendations in the Guide for the Care and Use of Laboratory Animals of the National Institutes of Health and in strict adherence with art. 18 of D.L. 26/2014 on organ and tissue sharing. (In order to reduce the number of animals used in procedures, the Ministry promotes the definition of programs, without new or increased burdens on public finances, for the sharing, among interested users, of organs and tissues from animals subjected to euthanasia for experimental purposes).

### Experimental design

The selected sheep (*N* = 20), 3-yr-old sheep weighing 45 ± 5.1 kg, were treated with a dose of the anthelmintic drug Ivermectin intramuscularly (Ivomec, Boehringer Ingelheim; 1 mL/50 kg body weight) to avoid any gastrointestinal parasites. In addition, the sheep underwent preoperative clinical, hematological, orthopedic, and ultrasound evaluations to exclude the presence of preexisting lesions on limbs.

The animals were placed in their barns at least 1 mo before the start of the study, in order to acclimatize themselves. They had free access to water and were fed fodder and concentrated food. Sheep were weighed before surgery and at regular intervals during the experimental period. Surgery was performed under aseptic conditions, and under both general and spinal anesthesia. All efforts were made to minimize suffering: all sheep were monitored daily in order to detect any alteration of the clinical conditions (food intake and weight loss; urine and feces production; rectal temperature and behavioral changes). All the sheep underwent radiographic evaluation (Aria Radiology, Foschi) at pre-established times: before surgery (time zero) and 2 wk after surgery (2w unloading). All the exams were exported as Dicom files and elaborated on horos software. The radiodensity of the tibia was analyzed using ImageJ software, manually outlining the tibia bone area.

At the end of the authorized experimental period and after euthanasia of the sheep, muscle biopsy samples were collected from the quadriceps femoris at the time of sacrifice. The first group of sheep (*N* = 8) was sacrificed 1 wk after surgery, and the second group of sheep (*N* = 12) was sacrificed 2 wk after surgery. The right limbs were always operated, while the left limb when unoperated was used as a control (contralateral). Briefly, muscle biopsy samples consisted of 10 unloaded hindlimb (HU) and 6 contralateral hindlimb at the 1-wk time point, and 19 HU and 5 contralateral at the 2-wk time point.

### Histological analysis of muscle

Quadriceps biopsies were immediately fixed in 4% paraformaldehyde solution (Sigma-Aldrich) for 24 h, then transferred to a 15% sucrose solution and subsequently stored in a 70% ethanol solution. Muscle specimens were dehydrated in increasing scales of ethanol and included in paraffin in cross position. Histological sections were cut with a thickness of 5 μm using a standard microtome (RM-2155 Leica). To perform staining procedures, paraffin was removed using Xylene solution and the sections were rehydrated in decreasing scales of ethanol. All reagents and dyes were purchased from Sigma-Aldrich (Sigma-Aldrich).

All sections were stained with Picro-Sirius Red Stain to quantify fibers cross-sectional area (CSA) or the percentage of collagen content in nonmyocyte area, following the manufacturer’s protocol.

Stained sections were digitalized using the whole-slide scanning platform Aperio ScanScope CS (Leica Biosystems) at the maximum magnification (40x) available and stored as high-resolution digital images on the workstation associated with the instrument. The images were analyzed using ImageJ software, manually outlining the perimeter of the individual muscle fibers to obtain the value of the CSA. The amount of collagen deposition was calculated as a percentage of the red-stained area with respect to the total area for each section using color-based thresholding with ImageJ software. At least 5 high power fields were analyzed for each section by 2 independent observers.

### Immunohistochemistry

Immunohistochemistry was performed on 5-μm-thick paraffin-embedded sections of quadriceps samples. After dewaxing, antigen retrieval was achieved with treatment with protease (Proteinase K; Roche) 0.1 mg/mL in PBS for 2 min at 37 °C. After a thorough rinse in phosphate buffered saline (PBS), sections were reacted with 0.3% H_2_O_2_ (in PBS; 30 min) to block endogenous peroxidase. Then, sections were incubated with the polyclonal rabbit anti-FNDC5 antibody (Abcam, Cat# ab181884) for 1 h at room temperature. After a thorough rinse in PBS, sections were incubated with biotinylated secondary antibody (AB2; Rabbit Specific HRP/DAB Detection IHC Kit, Abcam) at RT for 30 min; then with streptavidin alkaline phosphatase (Rabbit Specific HRP/DAB Detection IHC Kit, Abcam) at RT for 10 min; finally sections were incubated with chromogen DAB at RT for 5 min and then mounted in glycergel (Agilent Dako), then counterstained with hematoxylin (HHS32-1L, Sigma-Aldrich). For negative controls, the same procedure was followed replacing the primary antibody with pre-immune serum. All observations were performed after scanning stained sections by Aperio CS2 (Leica Biosystems) at the maximum magnification (40x). At least 5 high power fields were analyzed for each section. Staining was never observed when the primary antibody was omitted (Figure S1). The percentage of FNDC5 positive fibers was calculated by using color-based thresholding on ImageJ software.

### Real-time PCR

Quadriceps samples were homogenized with ultra-turrax T8 (Ika). Total RNA from sheep muscular tissue was extracted using spin columns (RNeasy, Qiagen) according to the manufacturer’s instructions. Reverse transcription was performed using iScript Reverse Transcription Supermix (Bio-Rad). The resulting cDNA (1 μg or 500 ng) was then subjected to quantitative PCR (qPCR) using the SsoFast EvaGreen Supermix (Bio-Rad) on Bio-Rad CFX96 Real-Time System (Bio-Rad) for 40 cycles (denaturation 95 °C for 5 s; annealing/extension 60 °C 10 s) after an initial 30 s step at 95 °C for enzyme activation. To verify the specificity of amplification products, melting curve was performed between 65 and 96 °C, with 0.5 °C incrementing every 10 s. Primers were designed by using Primer Blast online portal (https://www.ncbi.nlm.nih.gov/tools/primer-blast/). The primer sequences were as follows: *Gapdh* (S-TCGGAGTGAACGGATTTGGC, AS-CCGTTCTCTGCCTTGACTGT); *FNDC5* (S-CATCCAGGGTCAGAGTCCAG, AS-AACAGGACCACGACGATGAT); *MyHC1* (S-TACCCTGGGGATGAGTATGACTT, AS-CCAATCAAAGACCCTGCCTTG); *MyHC2a* (S-AGACATACCGTGGTTGGG, AS-CAGCACGCCGTTACACCT); *MyHC2x* (S-CCCTATAAAAGTACCCTGGGGATG, AS-CAAAGACCCTGCCTTGGAGA); *Murf1* (S-AGTCCTGGGAGGGGCATC, AS-TGTCAGCTACGTGCTCCAAG); *Atrogin* (S-CTTCTCCGAGCGGCAGATCC, AS-TGGGTAGCATCGCACAAGGTT); *NMRK2* (S-TACTTCCTGACCGTCCCCTA, AS-TACACCACTTCCAGCACCGTT); *PGC1alpha* (S-CCGTGCTCAGAGCTTCTCAA, AS-CTGCTGGTTCCGGTTCTCTGT); *Sirt1* (S-TGCTCGCCTTGCAATAGACT, AS-TCCACTGCACAGGCACATAC); and *mTOR* (S-AAGGTCTATTTGCCTCGCGGT, AS-AGCCTTCTGTCTCTTATGGGC). *Gapdh* was chosen as housekeeping gene, because it is stably expressed in sheep muscle. All primers span an exon–exon junction. Each transcript was evaluated in triplicate and quantitative measures were obtained according to the ΔΔCT method and expressed as a fold change compared to the control.

### Statistical analysis

All variables were checked for normality (Shapiro–Wilk normality test) to see the data distribution. For parameters with normal distribution, mean values were compared using Student’s *t*-test, otherwise, for parameters with non-normal distribution, significance was evaluated with Mann–Whitney test. For linear regression analysis, we calculated Pearson’s correlation coefficient for values with normal distribution, and the Spearman’s coefficient for parameters with non-normal distribution. The results were considered statistically significant if *p* values were ≤.05. GraphPad Prism 9.5 was used to perform statistical analysis and graph design.

## Results

**Figure 1:**
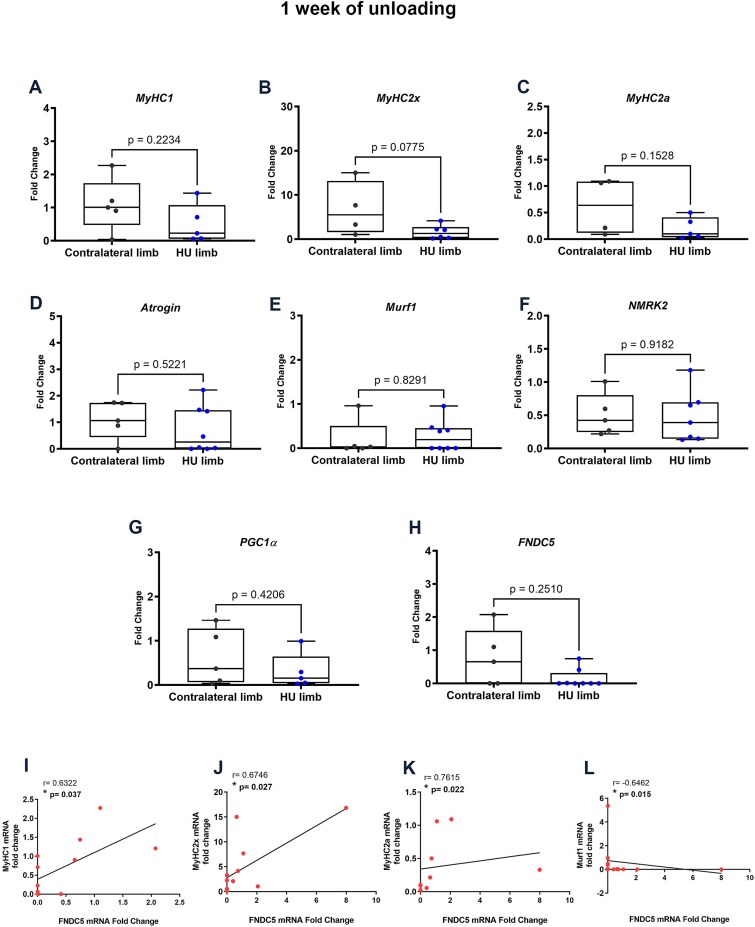
The effect of 1-wk unloading on the expression of myosins, FNDC5, atrophy, and regeneration markers in the skeletal muscle of sheep. Quantitative PCR (qPCR) analysis in the sheep quadriceps of HU limbs compared with the contralateral limb (CL) showed the expression levels of the myosin isoforms *MyHC1* (A) (CL *n* = 5; HU *n* = 5), *MyHC2x* (B) (CL *n* = 4; HU *n* = 6), and *MyHC2a* (C) (CL *n* = 4; HU *n* = 5), *Atrogin* (D) (CL *n* = 5; HU *n* = 8), *Murf1* (E) (CL *n* = 5; HU *n* = 8), *NMRK2* (F) (CL *n* = 5; HU *n* = 7), *PGC1*α (G) (CL *n* = 5; HU *n* = 5), and *FNDC5* (H) (CL *n* = 5; HU *n* = 9). Correlations of gene expression levels between *FNDC5* and the isoforms of myosin *MyHC1* (I), *MyHC2x* (J) and *MyHC2a* (K), and *Murf1* (L). Data are presented as box-and-whisker with median and interquartile ranges, from max to min, with all data points shown. Shapiro–Wilk test followed by Pearson or Spearman linear regression analysis for linear regression analysis, *r* and *p* values as indicated.

To examine the effects of unloading on quadriceps harvested from sheep, contralateral and hindlimb unloaded (HU) muscle biopsies were analyzed by real-time PCR. After 1 wk of unloading, we observed a trend toward reduced expression of type 1 myosin heavy chain (*MyHC1*), type 2X myosin heavy chain (*MyHC2x*), and type 2A myosin heavy chain (*MyHC2a*) in the HU quadriceps compared to the contralateral limb, although not significant. (Figure 1A-C). No changes in the expression of the Muscle Atrophy F-box gene (*Atrogin*), Muscle Ring-Finger Protein-1 (*Murf1*), and Nicotinamide riboside kinase 2 (*NMRK2*), which are involved in muscle atrophy and regeneration, were detected (Figure 1D-F). Additionally, we observed a trend toward downregulation of both *FNDC5* and its transcriptional coactivator peroxisome proliferator-activated receptor gamma coactivator 1-alpha (*PGC1a*) (Figure 1G-H). To determine whether there was a possible explanation for this concomitant down-regulation trend of FNDC5 and myosin isoforms, we performed linear regression analysis demonstrating the existence of a significant positive association between *FNDC5* fold-changes and *MyHC1* (Figure 1I), *MyHC2x* (Figure 1J), or *MyHC*2a (Figure 1K). Of note, we observed a negative correlation between *FNDC5* and *Murf1* levels, suggesting that higher *FNDC5* expression coincided with lower muscle atrophy markers (Figure 1L).

**Figure 2:**
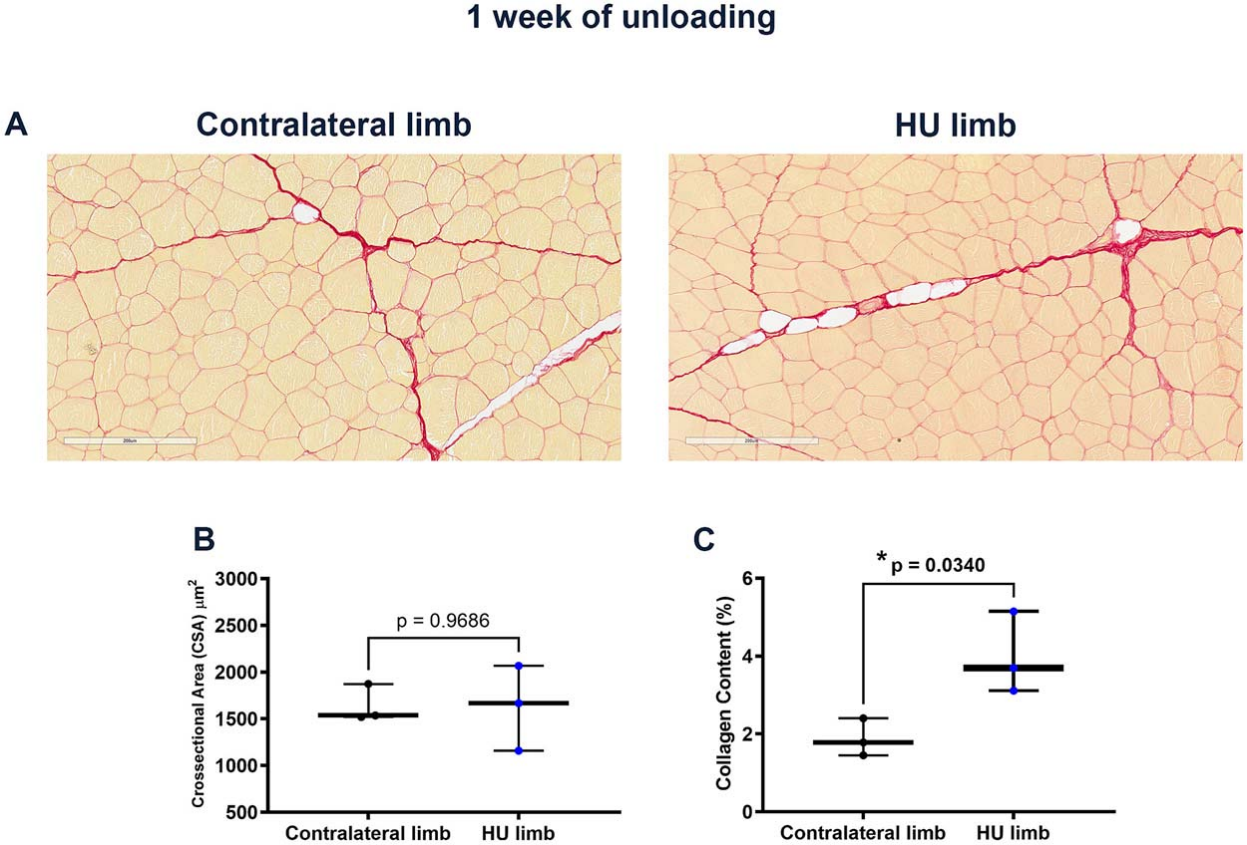
The effect of 1-wk unloading on the morphology of sheep muscle fibers. Representative images of Picrosirius Red staining in muscle biopsies of contralateral and HU limbs (magnification: 20x) (A). Morphometric analysis of cross-sectional area (CSA) of muscle fibers in contralateral (*n* = 3) and HU (*n* = 3) limbs (B). Quantitative assessment of collagen content percentage in contralateral (*n* = 3) and HU (*n* = 3) limbs (C). *p* value as indicated.

Picrosirius Red staining performed on transverse sections of quadriceps biopsies (Figure 2A) showed no differences in the cross-sectional area (CSA) of muscle fibers between contralateral and HU limbs (Figure 2B); however, the percentage of collagen content was significantly higher in the HU compared with contralateral limbs (Figure 2C), suggesting increased fibrosis in skeletal muscle of the operated legs after 1 wk of unloading.

**Figure 3:**
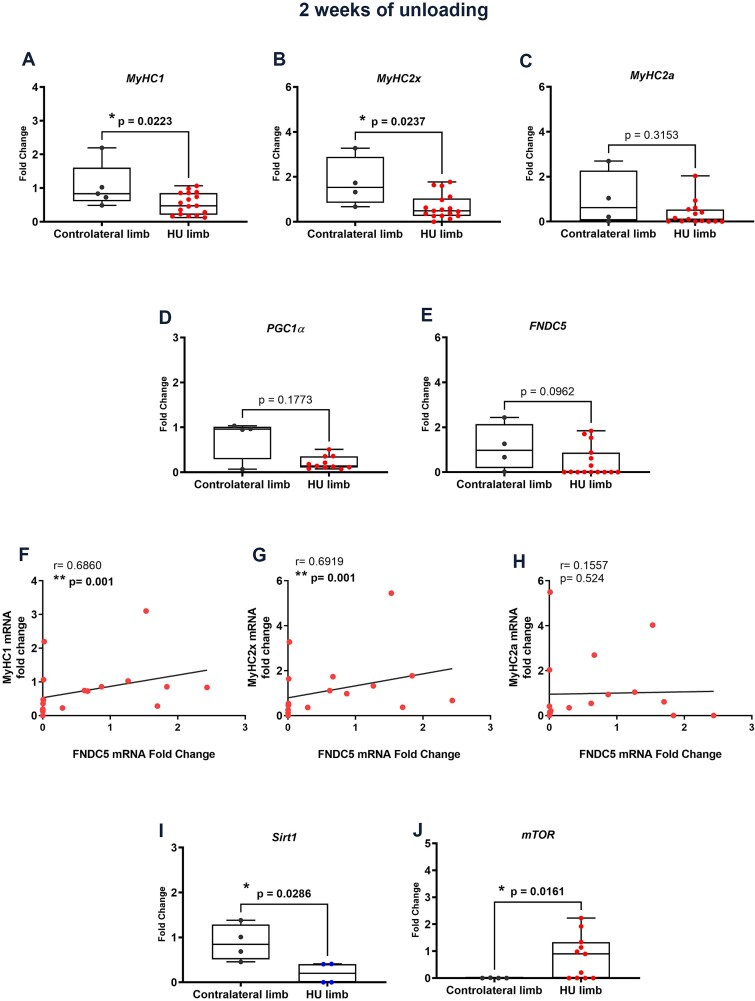
The effect of 2-wk unloading on the expression of myosins and FNDC5 in the skeletal muscle of sheep. Quantitative PCR (qPCR) analysis showed the expression levels of the myosin isoforms *MyHC1* (A) (CL *n* = 5; HU *n* = 17), *MyHC2x* (B) (CL *n* = 4; HU *n* = 17), and *MyHC2a* (C) (CL *n* = 4; HU *n* = 15), *PGC1*α (D) (CL *n* = 4; HU *n* = 11), *FNDC5* (E) (CL *n* = 4; HU *n* = 15), in the sheep quadriceps of HU limbs compared with contralateral limb. Correlations of gene expression levels between *FNDC5* and the isoforms of myosin *MyHC1* (F), *MyHC2x* (G), and *MyHC2a* (H). Quantitative PCR (qPCR) analysis in the sheep quadriceps of HU limbs compared with contralateral limb showed the expression levels of *Sirt1* (I) (CL *n* = 4; HU *n* = 4) at 1-wk unloading, and *mTOR* (J) (CL *n* = 4; HU *n* = 11) at 2-wk unloading. Data are presented as box-and-whisker with median and interquartile ranges, from max to min, with all data points shown. Shapiro–Wilk test followed by Pearson or Spearman linear regression analysis for linear regression analysis, *r* and *p* values as indicated.

We also examined quadriceps from sheep after 2 wk of unloading. Real-time PCR analysis showed that *MyHC1* (*p =* .0223) (Figure 3A) and *MyHC2x* mRNA (*p =* .0237) (Figure 3B) were significantly downregulated in HU limb compared with Contralateral ones, whereas *MyHC2a* (Figure 3C), PGC1α (Figure 3D), and *FNDC5* (Figure 3E) expressions showed a downward trend compared with the contralateral limb, albeit not significant (Figure 3C-E). No changes in the expression of the *Atrogin*, *Murf1*, and *NMRK2* were detected after 2 wk of unloading (data not shown). Interestingly, as already observed after 1 wk of unloading, the linear regression analysis showed a significant positive correlation of *FNDC5* fold-changes with *MyHC1* (Figure 3F) and *MyHC2x* (Figure 3G) confirming that higher *FNDC5* expression paired with higher synthesis of the major functional proteins of skeletal muscle. On the other hand, no correlation was seen between *FNDC5* and *MyHC2a* (Figure 3H). Notably, we also observed downregulation of Sirtuin 1 (*Sirt1*) mRNA (*p =* .0286) (Figure 3I) and an increase in mammalian target of rapamycin (*mTOR*) (*p =* .0161) (Figure 3J) in the quadriceps of sheep subjected to 1 and 2 wk of unloading, respectively, compared to the contralateral quadriceps.

**Figure 4:**
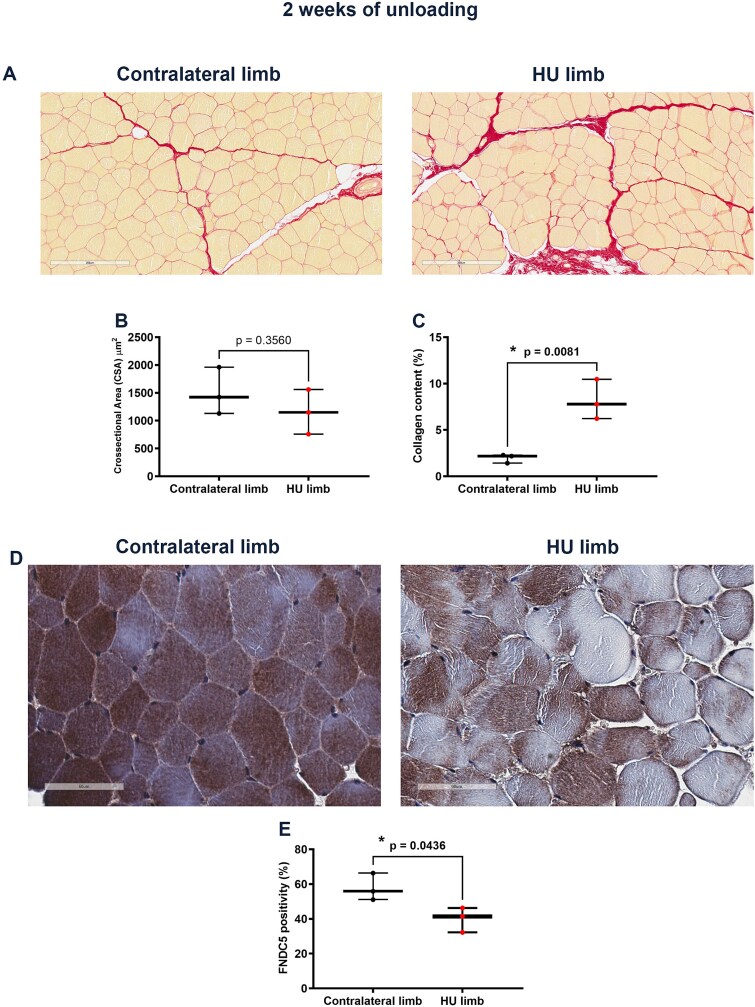
The effect of 2-wk unloading on the morphology of sheep muscle fibers. Representative images of Picrosirius Red staining in muscle biopsies of contralateral and HU limbs (magnification: 20x) (A). Morphometric analysis of cross-sectional area (CSA) of muscle fibers in contralateral (*n* = 3) and HU (*n* = 3) limbs (B). Quantitative assessment of collagen content percentage in contralateral (*n* = 3) and HU (*n* = 3) limbs (C). Representative images of immunohistochemistry staining of FNDC5 protein in muscle biopsies of contralateral and HU limbs (magnification: 40x) (D). Quantitative assessment of the percentage of FNDC5 positivity in muscle fibers from contralateral (*n* = 3) and HU (*n* = 3) quadriceps (E). *p* value as indicated.

After 2 wk of unloading, Picrosirius Red staining performed on transverse sections of quadriceps biopsies (Figure 4A) showed that muscle fibrosis became significantly higher in skeletal muscle of HU limbs compared with contralateral controls (Figure 4C), as evidenced by quantification of collagen content percentage; however, there was no detectable change of CSA (Figure 4B). Noteworthy, immunostaining on muscle fibers demonstrated significant reduction in the positivity for FNDC5 in HU limb compared with contralateral limb (Figure 4D-E), thus implying a strong impact of unloading on irisin precursor synthesis in the sheep’s muscle. To ensure that the 2 wk of unloading did not affect the bone, we performed X-rays of the femur and tibia, which showed unchanged radiodensity at 2 wk of unloading compared to preoperative X-rays (time zero) (Figure S2A and B). The result was quite expected since in our previous study performed on a mouse model, bone loss was observed starting from the fourth week of unloading.^2^^,^^4^

## Discussion

The results of this study showed that the precursor of irisin, FNDC5, and 2 of the main functional proteins of skeletal muscle, the myosin isoform *MyHC1* and *MyHC2x*, are negatively affected by the absence of mechanical load in sheep quadriceps, as already demonstrated in murine models.^2^^,^^4^ In addition, a positive correlation between *FNDC5* and myosins gene expression was also observed, suggesting that the irisin/FNDC5 system is a driving force for muscle function also in sheep, a large animal model.

There is only one study that has demonstrated the expression of *FNDC5* in sheep muscle, identifying it through transcriptomic analysis as 1 of 6 possible molecular marker genes related to production performance and meat quality for molecular selection of sheep.^11^ The results of our study add another piece to the existing knowledge about FNDC5 expression providing new evidence that it is sensitive to mechanical loading also in ovine animals.

Surprisingly, our data show that the downregulation of *FNDC5* in unloaded quadriceps is not accompanied by an equally significant reduction in its co-activator *PGC1α*; rather, the latter showed only a trend toward reduction. Although PGC-1α is widely recognized as one of the main regulators of FNDC5 expression in skeletal muscle during exercise, it is not the only activator. Recent studies indicate that other pathways and factors also regulate FNDC5 transcription and expression.^12–14^

We observed a progressive decrease in the gene expression of *MyHC1* and *MyHC2x*, starting with a downward trend reduction after 1 wk of unloading, and finally reaching a significant reduction at 2-wk compared to contralateral muscle. This effect mirrors what we had already observed in the HU mice, in which the downregulation of *MyHC2x* was already significant after 1 wk of unloading.^2^ One possible explanation for the different timing of gene regulation could be that the negative effects of unloading are delayed in the transition from rodents to large animals.

Our findings also showed that *Sirt1* expression is reduced, but, surprisingly, mTOR is upregulated in the unloaded quadriceps compared to their contralateral counterparts. As for the downregulation of *Sirt1* mRNA, this result was predictable, given that sirtuins, particularly Sirt1, play a crucial role in regulating muscle atrophy by modulating pathways related to protein degradation and muscle waste.^15^ Moreover, *Sirt1* is known to be involved in the production of FNDC5/irisin during exercise,^16^ while little is known about the effect on *Sirt1* in the absence of mechanical loading. With regard to the unexpected upregulation of mTOR, a recent review has highlighted that a common misconception in scientific literature is the belief that, since protein synthesis is suppressed under conditions that induce muscle atrophy, the mTOR signaling pathway is consequently inhibited as well.^17^ However, under conditions of immobilization, it has been reported that mTOR activity increases, rather than decreases, at least during the early stages of the atrophy process.^18^^,^^19^

On the other hand, compared to the mouse model of HU, in the quadriceps of sheep we found no alterations in the expression of atrophy marker genes, such as *Atrogin* and *Murf1*, and no reductions in muscle fiber CSA. However, evidence of activation of the ubiquitin-proteasome system by Atrogin and Murf1 during muscle disuse appears to depend on the temporal course of the atrophy process.^20^ Previously, an increase in these markers has been observed in vastus lateralis biopsy samples after 2 d of immobilization in humans.^21^ However, some studies have found no change or even observed a decrease in the expression of Atrogin and Murf1 after periods of disuse of 14 d or more, while others have shown a transient induction of these atrophy marker genes.^22^ As regards the undetectable reduction in CSA of muscle fibers in sheep quadriceps subjected to unloading, we believe that, in this case as well, an unloading period of more than 2 wk would be necessary to observe muscle atrophy. However, the morphological data from the present study showed a marked increase in muscle fibrosis that was already significant after 1 wk of unloading. Numerous studies have shown that muscle fibrosis can cause more severe damage to muscle function than reduction in fiber area.^23^ In particular, the high regenerative capacity of skeletal muscle can be severely compromised during unloading by excessive cellular matrix deposition, leading to the onset of muscle fibrosis.^24^ This excessive deposition of fibrous tissue not only compromises muscle function^25^ but also increases the muscle’s susceptibility to new injuries and can slow down muscle fiber regeneration.^26^ Our results showed that fibrosis develops earlier than muscle atrophy in the absence of mechanical load in sheep. It will be essential in the future to focus on understanding the mechanisms that trigger muscle fibrosis, as this could be relevant for the prevention and treatment of sarcopenia in humans caused by immobilization, as occurs in elderly or bedridden individuals. In this regard, sheep appear to be an excellent large animal model for studying the musculoskeletal system, since it has been demonstrated that the distribution of mechanical loads acting on the joints during loading activities is similar to humans.^10^^,^^27^

Our study has some limitations; in particular, it was conducted only on the quadriceps muscle, which is composed of mixed muscle fibers. Most of the studies have been conducted in HU mouse models on the soleus muscle, an antigravity muscle that is particularly sensitive to unloading, in which an early and transient increase in *Atrogin* and *Murf1* mRNA levels was observed as early as 3 d of HU.^28^ Moreover, studies conducted on astronauts after six months aboard the International Space Station have also shown a shift from slow-twitch fibers to fast-twitch fibers in the soleus muscles.^29^ Therefore, to confirm the suitability of the sheep as a large animal model for studying the effects of unloading on skeletal muscle, data on muscles with a predominance of type 1 muscle fibers, such as the soleus muscles, would have been useful. Another limitation is the constraint imposed by the 2-wk duration of the mechanical unloading period, a timeframe that may have been insufficient to allow for an assessment that goes beyond the expression of FNDC5.

We believe that the results of this study, despite some limitations, can improve the translational pathway. In fact, the translation of drugs from the preclinical phase conducted in rodent models to the clinical phase has sometimes been unsuccessful, partly due to the choice of experimental model. The sheep model, unlike the widely adopted rodent model, has the advantage of improving musculoskeletal translation, particularly due to the body size, long lifespan, and similarities in anatomical structures of sheep to humans. However, it is important to emphasize that, although sheep have proven to be relevant for the study of drug administration by injection or inhalation, there would be limitations for the study of oral drug administration, due to different digestive systems of ruminants compared to humans.

## Supplementary Material

Supplementary_Materials_file_ziag057

## Data Availability

Data are available in a publicly accessible repository that does not issue DOIs. Data can be found here: Raw data_Suriano-Terrevoli et al accessed on March 16, 2026.
