# Supplementary material for: Impact of mechanical unloading on the irisin precursor, FNDC5, in skeletal muscle of sheep as a large animal experimental model
Source: JBMR Plus. 2026 Mar 31;10(5):ziag057. doi: 10.1093/jbmrpl/ziag057 (PMC13117608; doi:10.1093/jbmrpl/ziag057)
Supplement: Supplementary_Materials_file_ziag057 [file supplementary_materials_file_ziag057.pdf]

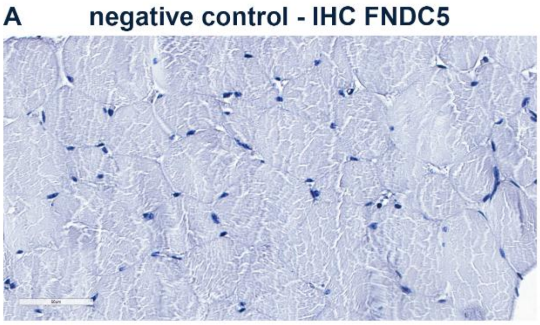

Supplementary Figure S1 Representative image of the negative control staining for FNDC5 in sheep skeletal muscle (counterstaining with hematoxylin) (**A**).

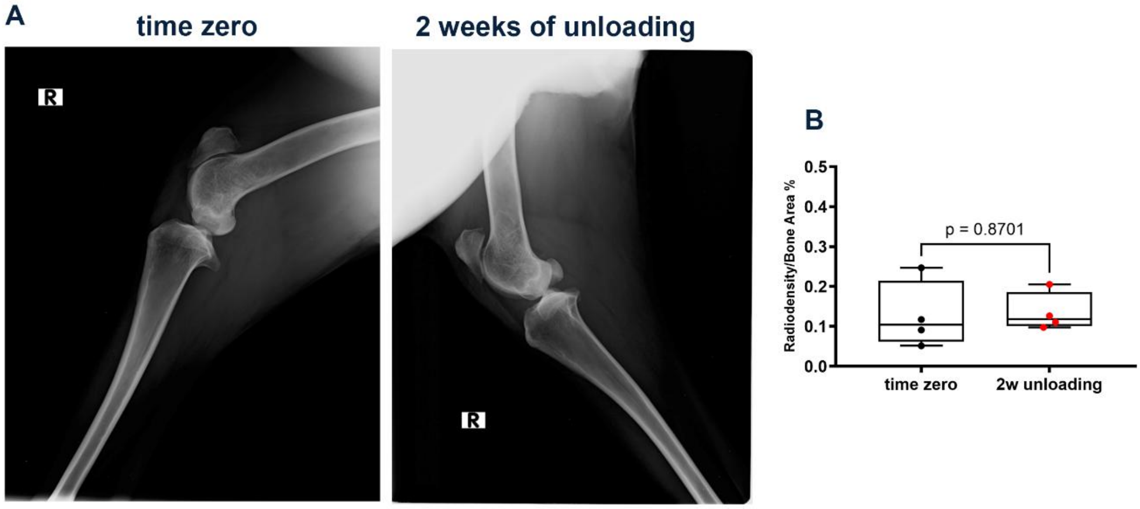

Supplementary Figure S2 The effect of 2-week unloading on bone. Representative images of radiographs of selected long bones at time zero and at 2 weeks of unloading (**A**). Morphometric analysis of radiodensity per bone area of right (R) HU limbs (**B**).
